# On the Form of the Face and Forehead; with a Theory of Their Formation in Different Ages and Nations

**Published:** 1829-04-01

**Authors:** 


### XXII.

### ON THE FORM OF THE FACE AND FOREHEAD; WITH A THEORY OF THEIR FORMATION IN DIFFERENT AGES AND NATIONS. By DR. MILLIGAN.*

The figure of the face and forehead must, of course, depend upon that of the bones beneath, but the *mode* in which these are developed, and the relation which their development bears to that of other bones of the body form the subject of Dr. Milligan's inquiry. Our ingenious author maintains that the evolution of the facial bones is governed by the following general laws:—

1. The external and internal plate of a flat bone are two different, distinct organs, merely connected with each other by diploe, as muscles are by cellular membrane, but each having an office corresponding to the organs contiguous to it.

2. Wherever those contiguous parts follow different laws of growth, the quicker growing bone separates from the slower, and leaves a space which is *filled up* by diploe, if no mucous membrane is near, and lined by mucous membrane, when that tissue is in the vicinity.

3. That this membrane prevents the formation of diploe or cancelli, and, consequently, *is the immediate cause* of those sinuses that excavate the bones of the face.

4. Dr. Milligan proves, that the difference between the growth of the brain, which stops at the 7th year, and the growth of the face bones, which stops not till the 21st year, is in every case the cause of such separation, admission of mucous membrane, and formation of facial sinuses.

Hence nations or individuals that have the brain soon brought to the full growth, must have these facial sinuses large and the cheek bones wide, and the reverse. Hence, also, the large heads and small faces of Hydrocephalus are easily explained.

We subjoin the passage from the original work, which is now furnished with a copious alphabetical index, and some valuable plates and tables.

"The development of the internal table of the skull, and, consequently, of the *frontal bone, follows the development of the brain; but the development of the external table of the frontal bone follows the development of the bones of the face.* Now, the brain, we have seen, arrives at its full size in the seventh year; which,

---

* We have just received Dr. Milligan's third edition of Magendie's Physiology, greatly enlarged and improved by notes, engravings, and an alphabetical index. We hasten to notice the above subject, which is a novelty.

therefore, is the period of completing the development of the internal table of the frontal bone. But the bones of the face continue growing to the twenty-first year; and hence it is, that anatomists find the dimensions of the frontal sinus go on increasing to that year; and the same authors *generally* find the sinus commence at the seventh year, because that is the time at which the nutritious arteries of the internal table cease to do more than support its vitality. Suppose that the day, or week, or month, after this has happened, a *line* in length, and a *line* in breadth is about to be added to the external table by the arteries, in order to preserve its congruity with the transverse suture, which is still growing. It is manifest, that the arteries which make this new interposit must lay down their bony matter a line farther forward than before; while the absorbents, whose modelling action necessarily accompanies deposition, will remove that part which was in contact with the diploe from the now fixed vitreous table, in which no corresponding tendency to continue the union by diploe will be excited, even should points of diploe be thrown out by the still continued formative efforts of the vessels of the external table. Nay, these efforts soon must become abortive from another cause. Bichat has shown that the cells of diploe are without any membraneous lining—its plates are themselves bony membranes. When, therefore, the arteries of the anterior table advance its position forwards, to make it coincide with the nasal bones, an opening is offered behind, into which creep the vessels of the Schneiderian membrane which is in immediate contact at this point, irritated by the change of position, and at the same time equally rapid in their depositing action with the vessels of the external table. Hence, a membrane is rapidly shot into the nascent hollow, or *sinus*, which attaching itself to the outer aspect of the vitreous table, and to the inner aspect of the osseous table, forms an insurmountable obstacle to the rudest diploe that might join these two layers. For it is a mucous membrane, a class which scarcely ever forms adhesions, and is here almost a shut sac, whose sides are every day brought farther and farther asunder.

"I would rather say, that it is a similar penetration of this mucous membrane that forms the other cavities in the bones of the face, than that this cavity of the forehead is formed according to the model and for the same uses as those of the face, though both views are in some measure true. These cavities augment the sound of the voice, and, by rendering the bones hollow, equipoise the occipital prolongation so exactly upon its condyles that the weight of a small coin is often sufficient to turn the balance. To man alone are given posterior lobes of the brain, and he alone requires a projection of the anterior lobes, and corresponding facial bones, to balance the former—a state rendered doubly necessary by his erect position; but had those facial bones been *solid*, a useless weight of three or four pounds would have been superadded, producing a most injurious preponderance, and defeating the ends of nature.

"Thus, then, we have the true theory of the frontal sinus, and it becomes easy to explain what have hitherto been named anomalies."

---

